# Mutation spectrum of Egyptian children with cystic fibrosis

**DOI:** 10.1186/s40064-016-2338-7

**Published:** 2016-05-20

**Authors:** Walaa Aboulkasem Shahin, Dina Ahmed Mehaney, Mona Mostafa El-Falaki

**Affiliations:** Department of Allergy and Pulmonology, Children’s Hospital, Cairo University, Cairo, Egypt; Department of Clinical and Chemical Pathology, Cairo University, Cairo, Egypt

**Keywords:** CFTR mutations, Cystic fibrosis, Egypt, F508del

## Abstract

**Objective:**

To know the common CFTR mutations in the Egyptian patients with cystic fibrosis as it was previously thought to be uncommon disease in Egypt.

**Methods:**

This is a cross sectional study of 60 patients diagnosed as cystic fibrosis by sweat chloride testing. They were enrolled from the Allergy and Pulmonology Unit Children’s Hospital Cairo University. They were screened for the presence of the frequent 36 mutations in Caucasians by reverse hybridization line probe technique, using INNO-LiPA*CFTR*19 and *CFTR*17 + Tn kits.

**Results:**

Most of patients presented with classic manifestations of CF such as variable pulmonary disease and pancreatic insufficiency, and hepatomegaly with or without ascites. The mutations detected were F508 del (58 %), 2183AA/G (10 %), N1303K (6 %), I148T (4 %), W1282X (4 %), G155D (2 %), CFTRdel2-3 (21 KB) (2 %), 3199del6 (2 %), R347P (2 %). Unique to the Egyptian population are these mutations R1162X and A544E (6, 4 %) respectively they were found in our cohort study and were not reported elsewhere in the Arab population till now. There was no association between the initial clinical presentation of CF and the genotypes detected.

**Conclusion:**

The F508 del is still the most commonly encountered mutation (58 %), however other rare mutations were identified where each ranged from (2 to 10 %).

## Background

Cystic fibrosis (CF), the most common lethal genetic disease in whites, affects approximately 70,000 people worldwide (Bethesda 2009). The basic defect in CF cells is the faulty chloride transport, which causes dehydration of secretions with hyper viscous mucus and leads to chronic airway obstruction, exocrine pancreatic insufficiency and intestinal malabsorption. While many organs are affected in CF, pulmonary disease is the major cause of morbidity and mortality (Lubamba et al. 2012).

Persistent respiratory symptoms, bronchiectasis, or clubbing should prompt a diagnostic evaluation for CF. Although nasal polyps can be seen in other conditions; their presence should suggest the possibility of CF (Voter and Clement 2008). The accurate knowledge of CFTR (cystic fibrosis Transmembrane conductance regulator) mutations is of obvious interest in clinical testing, as it improves CF prevention programs of neonatal screening, heterozygote screening in partners of CF patients or partners of carriers. Reporting updated data specifically for each group of populations is also crucial for deeper understanding of CF genetics (Des Georges et al. 2004).

CFTR mutations vary in their frequency and distribution in different populations. CF was previously thought to be rare among Arabs, however there are some published data denoting its existence (Banjar 1999; Farra et al. 2010; Siryani et al. 2015).

Our aim in the present study was to detect the common mutations encountered in a group of Egyptian CF population, using the commercially available kits. Better understanding of the genetic background of our population and its relation to the clinical presentation of CF, will allow us for providing optimum care for our patients.

## Subjects and methods

### Subjects

This pilot study included 60 CF patients enrolled from to the CF clinic of the Allergy and Pulmonology Unit, Children’s Hospital Cairo University over a period of 2 years (January 2013–January 2015); where these patients came regularly for follow up and receiving medications. Patients were diagnosed as having CF based on clinical presentations and confirmed by sweat chloride testing, which was repeated twice as per the cystic fibrosis foundation (CFF) guidelines (Le Grys et al. 2007). Blood samples were withdrawn for genetic testing of the frequent 36 mutations in Caucasians (Lenarduzzi et al. 2014).

### Quantitative sweat chloride testing

Sweat stimulation was done using the pilocarpine iontophoresis for induction and sweat collection by the Wescor macroduct sweat collection system (NCCLS 2000). The sweat sample was analyzed quantitatively by the thiocyanate colorimetric method (Skeggs and Hochstrasser 1964). Test result higher than 60 mmol/L was considered positive and consistent with the diagnosis of CF.

### Molecular analysis

DNA was extracted from whole blood samples using QIAamp Mini Kit (QIAGEN, USA). All patients with positive sweat chloride test were screened for the presence of the 36 frequent mutations in Caucasians, using INNO-LiPA*CFTR*19 and *CFTR*17 + Tn kits (Innogenetics, Ghent, Belgium). This is a multi parameter line probe assay based on the reverse hybridization principle for the simultaneous detection and identification of 36 CF-related mutations and their wild-type sequences in human whole blood.

Briefly, multiplex PCR was done in a reagent mix containing biotinylated primers, dNTPs and hot start Taq polymerase. Amplification was done according to the manufacturer’s protocol: 30 cycles of denaturation at 95 °C for 1 min, annealing at 57 °C for 1 min, extension at 68 °C for 1 min, then final extension at 68 °C for 10 min. Amplified biotinylated DNA was chemically denatured and hybridized with specific oligonucleotide probes immobilized on membrane-based strips. Streptavidin conjugated with alkaline phosphatase was added to bind to any biotinylated hybrid formed. Incubation with a substrate solution containing a specific chromogen resulted in a pink color and positive patterns were recorded.

### Statistical methods

Data was analyzed using IBM SPSS (software package used for statistical analysis) Advanced Statistics version 20.0 (SPSS Inc., Chicago, IL). Numerical data were expressed as mean and standard deviation (SD) or median and range as appropriate. Qualitative data were expressed as frequency and percentage.

## Results

The present study enrolled 60 Egyptian children with CF, their age ranged from 6 months to 14 years. Thirty eight of them were males and 22 were females. Positive consanguinity was reported in 57 % of patients. 23 % of patients had positive family history of CF; the most frequent clinical presentations were pulmonary disease 84 % followed by pancreatic insufficiency (56 %). Demographic and clinical data of the studied population are summarized in Table 1.

**Table 1:** Demographic data and clinical presentations of the study population

Variables	N = 60 (%)
Age (years)^a^	5 (0.5–14)
*Gender*	
Male	38 (63)
Female	22 (37)
Positive family history of CF	14 (23)
Positive consanguinity	34 (57)
*Clinical presentations*	
Pulmonary disease	56 (93)
Pancreatic insufficiency	52 (87)
Hepatic disease	9 (15)
Intestinal obstruction	2 (3)
Nasal polyps	13 (22)
Sinusitis	13 (22)
Pseudo barter	1 (1.66)

Data are presented as number (percent)

^a^Data are presented as median (range)

Twenty seven out of the 60 patients proved to be positive for the 36 mutations searched for. Among the 120 alleles examined, 50 positive alleles (41.6 %) were detected as shown in Table 2.

**Table 2:** Frequency of mutations detected among the studied patients

Mutation	Allele frequency, N = 50 (%)	Class of mutation
ΔF508	29 (58)	Class II Non-functional, degraded mutant CFTR protein
2183AA/G	5 (10)	Class I Shortened protein
N1303K	3 (6)	Class II
R1162X	3 (6)	Class IV Reduced chloride conductance
I148T	2 (4)	Alone it is a neutral polymorphism not causing CF
W1282X	2 (4)	Class I Shortened protein
A544E	2 (4)	
G155D	1 (2)	Class III Channel cannot be regulated properly
CFTRdel2,3 (21 KB)	1 (2)	Class I Shortened protein
3199del6	1 (2)	Class II
R347P	1 (2)	Class IV Reduced chloride conductance

Data are presented as number (percent)

The most commonly encountered mutations were F508del (58 %) followed by 2183AA/G (10 %). Testing of intron 8 (T) n variants revealed that the most frequent allele was the T7 allele (71.7 %) followed by the T9 allele (25.8 %) and the T5 allele (2.5 %). The mean ± (SD) age of patients with positive mutational testing was 6.7 (4.3) years. Male:Female ratio was 2.6:1. Most of patients (24/25) presented with pulmonary disease and pancreatic insufficiency. The mean (SD) sweat chloride concentration was 87.5 (12.5) mmol/L; ranging from 69 to 110 mmol/L. Positive consanguinity was present in 57 % of patients. Positive family history was present in 23 % of cases. Results of sweat chloride concentrations; genotypes and phenotypes of the 25 positive patients with are given in Table 3.

**Table 3:** Demographic, phenotype and genotype characteristics of the 27 patients with positive mutations

Pt No	Sex	Age (Y)	Genotype	T repeats	Sweat chloride	Phenotype
1	M	2	Hetero, R347P	T7	79	PS/Pul. Dis.
2	F	3	Homo, 2183AA/G	T7	69	PI/Pul. Dis.
3	F	4	I148T/3199del6	T7/T9	104	PI/Pul. Dis., *Rectal prolapse*
4	M	6	Hetero, N1303K	T7	71	PI/Pul. Dis.
5	M	7	Homo, DeltaF508	T7	95	PI/Pul. Dis.
6	M	3	Homo, N1303K	T7	70	PI/Pul. Dis.
7	M	11	Homo, DeltaF508	T7	104	PI/Pul. Dis./Nasal polyps/sinusitis
8	M	4	Homo, DeltaF508	T7	88	PI/Pul. Dis./Hepat. Dis.
9	F	14	DeltaF508/W1282X	T7	90	PI/Pul. Dis.
10	M	5	2183AA/G/R1162X	T7	99	PI/Pul. Dis./Nasal polyps
11	M	17	DeltaF508/W1282X	T7	100	PI/Pul. Dis.
12	F	3	Homo, DeltaF508	T7	76	PI/Pul. Dis.
13	M	5	Homo, DeltaF508	T7	105	PI/Pul. Dis.
14	F	8	Homo DeltaF508	T7	102	PI/Pul. Dis./Nasal polyps/sinusitis
15	M	7	Homo, DeltaF508	T7	85	PI/Pul. Dis.
16	M	17	2183AA/G/R1162X	T7	75	Nasal polyps/sinusitis
17	F	5	Homo, DeltaF508	T7	70	PI/Pul. Dis./Hepat. Dis.
18	F	5	Hetero, CFTR del 2,3 (21 kb)	T7	75	PI/Pul. Dis.
19	M	8	Homo, DeltaF508	T7	82	PI/Pul. Dis./sinusitis
20	M	8	Homo, DeltaF508	T7	80	PI/Pul. Dis./Nasal polyps/sinusitis/Hepat. Dis.
21	F	6	I148T/AA2183/G	T7	95	PI/Pul. Dis.
22	M	8	Homo, DeltaF508	T7	100	PI/Pul. Dis./Nasal polyps/sinusitis/Hepat. Dis.
23	M	4	DeltaF508/R1162X	T9	90	PI/Pul. Dis./Hepat. Dis
24	M	3	Homo, A455E	T7	75	PI/Pul. Dis.
25	F	1	Hetero, G551D	T7	110	PI/Pul. Dis./*Pseudo Barter syndrome*
26	M	5	Homo, DeltaF508	T7	80	PI/Pul. Dis./Nasal polyps/sinusitis
27	M	14	Homo, DeltaF508	T7	95	PI/Pul. Dis./Nasal polyps/sinusitis

*Y* yes, *N* no, *Pt* patient, *Y* years, *M* male, *F* female, *FH* family history, *Pul. Dis.* pulmonary disease such as chronic cough, wheezing or recurrent bronchitis, *PI* pancreatic insufficiency, *PS* pancreatic sufficiency, *Hepat. Dis.* hepatic disease such as hepatomegaly, ascites. T7, T5 and T9 refer to the allele variants commonly known as IVS8 Tn repeats. *Homo* homozygote, *Hetero* heterozygote

## Discussion

Although the most common CFTR mutation is F508del, which accounts for 65–85 % of all reported CFTR mutations in Caucasian populations, New and novel mutations have been detected in the Egyptian CF population as well as the different Arab communities who carry CFTR mutations that had never been described in the Caucasians (Farra et al. 2010). Limited data are available about CF in Egypt. Only two recently published articles highlighted the presence of many cases assuming that it is not uncommon in Egypt (Naguib et al. 2007; El-Falaki et al. 2014).

Our study addressed the most common CFTR mutations in another 60 CF patients different from those included in our previous study (El-Falaki et al. 2014). Most of patients included in the sample had typical clinical manifestations of repeated chest infection, variable pulmonary disease, steatorrhea, failure to thrive and early liver disease. The mutations detected were F508 del (58 %), 2183AA/G (10 %), N1303K (6 %), I148T (4 %), W1282X (4 %), G155D (2 %), CFTRdel2-3 (21 KB) (2 %), 3199del6 (2 %), R347P (2 %). The F508 del represented (58 %) of the mutations detected in our study population, this was in near frequency to what was found in United Arab Emirates and Tunisia where F508del represents 94 and 50.74 % respectively (Frossard et al. 1998; Messaoud et al. 2005). Where as in other populations like Iran F508del was found in only 18 % (Alibakhshi et al. 2008).

Our patients who were homozygous for theF508del had severe pulmonary disease, severe PI and liver disease. These finding were in accordance with Castellani et al. (2008) who showed that Patients homozygous for F508del usually have more pronounced clinical manifestations compared to compound heterozygotes and genotypes without F508del, although these differences are highly variable. Patients homozygous for the F508del mutation have an earlier diagnosis of disease, higher sweat chloride levels, younger age at presentation and are more likely to be pancreatic insufficient (Castellani et al. 2008).

The initial presentation of one female patient was pseudo Bartter’s syndrome (PBS) at the age of 6 month who later developed chest infection and steatorrhea. She was heterozygous for G551D mutation, which is the third most common CF-causing mutation, and is present in about 4 % of patients (Kotha and Clancy 2013), it was found in United Kingdom and central Europe, Syria and Egypt. We report here for the first time in the literature this mutation in a case of PBS. Another previously reported mutation in one patient with CF and presenting with PBS, severe dehydration and hypochloremic metabolic acidosis in 2011 were (*3849* + *1G*>*A and 4382delA compound heterozygosity*) (Nahida et al. 2011), this was in addition to *M2789* + *5G and F508del* described in 2013 in Jordan (Dahabreh and Najada 2013) and *D575G* mutation in southern Italy (Salvatore et al. 2004).

The mutation *2183AA/G* was encountered in 10 % of our studied alleles. It is a frameshift mutation (A to G at 2183 and deletion of A at 2184). It was encountered in similar frequency in Syria and Algeria (Jarjour et al. 2015; Loumi et al. 2008). It was also found in Southern Europe, Iran, and Latin America (Rolfini and Cabrini 1993). Patients who have this mutation had classic clinical manifestations of variable pulmonary disease and pancreatic insufficiency which agreed with other studies suggesting that this phenotype could lead to moderate–severe symptoms (Pereira et al. 1999). The mutations *R1162X* and *A544E* are present in the Egyptian population in a ratio of 6 and 4 %, respectively. To our knowledge they were not described elsewhere in the Arab population till now.

The mutation *R1162X* was detected in three patients; two were heterozygotes for 2183AA/G/R1162X, but with variable pulmonary and pancreatic involvement, recurrent nasal polyps and sinusitis. However when this mutation *R1162X* comes with F508 it leads to more severe symptoms in addition to liver affection. This is a very common nonsense mutation in north eastern Italy which usually presents with mild to moderate pulmonary disease and severe pancreatic insufficiency (Rolfini and Cabrini 1993).

The mutation A544E was detected in one patient as homozygous mutation, this patient had mild pulmonary disease and PI with failure to thrive. This exceedingly rare A455E mutation (identified in only eight patients in the United States) is the best described CF-causing mutation associated with a mild CF lung phenotype (Walker et al. 1997). Two Patients having the *N1303K* mutation either homozygous or heterozygous have the same classic clinical presentation of pulmonary affection and pancreatic insufficiency. This was in contrast to Van Hoorenbeeck et al. (2007) who found that this mutation was associated with mild atypical CF disease presenting at variable ages (Van Hoorenbeeck et al. 2007).

The mutation *I148T* was found in a compound heterozygotes form with *2183AA/G* in one patient and with 3*199del6* in another patient who had rectal prolapse in addition to the classic CF pulmonary disease and pancreatic insufficiency. This was in accordance to the study done in 2004 declaring that *I148T* exists as a complex allele with *3199del6* in patients with clinical CF and recommended that Reflex testing for *3199del6* should be considered whenever *I148T* is identified (Monaghan et al. 2004). The mutation W1282X detected in our study was also found in other Arab countries like Lebanon, Palestine, Syria, Tunisia and Algeria (Farra et al. 2010). However, it showed a higher frequency than F508del in some populations such as Ashkenazi Jews (Shoshani et al. 1992).

CF patients in our study bearing the W1282X mutation were in compound heterozygote form with F508del. They have severe pulmonary and pancreatic disease, this was in accordance with Rolfini and Cabrini in 1993 who found that CF patients bearing W1282X present severe pulmonary and pancreatic disease, whereas patients carrying other nonsense mutations such as G542X, R553X, S1255X, R1162X, and W1316X show a severe pancreatic but mild pulmonary illness (Rolfini and Cabrini 1993).

Our study showed that the mutation R347P *was* detected once (2 %) in a compound heterozygous form with unknown other mutations. It occurs with an overall worldwide frequency of about 0.2 %. This patient had mild pulmonary disease and pancreatic sufficiency, which differs from what Vron et al. found during studying a group of 19 CF patients with this mutation of German, Bulgarian, Czech, and Slovak origin; where most patients presented with early disease onset, pancreatic insufficiency and early pulmonary involvement, suggesting that this mutation can lead to a severe course of CF (Varon et al. 1995).

The deletion mutation CFTRdel2,3 (21 KB) was found in a compound heterozygous form with unknown other mutation in another patient with moderate to severe pulmonary disease and pancreatic insufficiency. These finding agreed with Dork et al. who found that this deletion represents a severe mutation associated with pancreatic insufficiency and early age at diagnosis, and that the 21-kb deletion is a frequent and severe CF mutation in populations of Eastern- and Western-Slavic descent (Dork et al. 2000).

## Conclusion

This study addressed the CFTR mutations in Egyptian patients with cystic fibrosis. The F508 del is the most commonly encountered mutation, yet there are some mutations that haven’t been described before. Genotype-phenotype correlation is not always easy to establish; genetic explanations are still being sought for different clinical manifestations in individuals with identical mutations, although the explanation may lie in interplay of genetic, infectious, and environmental factors. DNA sequencing is required in order to detect further mutations that will provide the basis for neonatal screening program as well as prenatal diagnosis.

What is already known?

Cystic Fibrosis is less common in Egypt and may not be considered in the differential diagnosis by many physicians. Profile of CFTR mutations for Egyptians remains unknown.

What this study adds?

The commonest mutation detected in the Egyptians with CF was ∆ F508 del (58 %), followed by other mutations 2183AA/G (10 %), N1303K (6 %), I148T (4 %), W1282X (4 %), G155D (2 %), CFTRdel2-3 (21 KB) (2 %), 3199del6 (2 %), R347P (2 %).

### Limitations of the study

- Limited mutation screening panel of 36 mutations only was used. It is now known that there are more than 2000 CFTR mutations.
- It represents single center data and may not reflect the prevalence of these mutations in the general Egyptian population.

## Abbreviations

CF: cystic fibrosis
CFTR: cystic fibrosis transmembrane conductance regulator
CFF: cystic fibrosis foundation
